# Recognizing non-native spoken words in background noise increases interference from the native language

**DOI:** 10.3758/s13423-022-02233-7

**Published:** 2022-12-21

**Authors:** Florian Hintz, Cesko C. Voeten, Odette Scharenborg

**Affiliations:** 1 Max Planck Institute for Psycholinguistics, P.O. Box 310, 6500 AH Nijmegen, The Netherlands; 2 Fryske Akademy, Leeuwarden, Netherlands; 3 Multimedia Computing Group, Delft University of Technology, Delft, Netherlands

**Keywords:** Bilingual processing, Onset competition, Eye-tracking

## Abstract

Listeners frequently recognize spoken words in the presence of background noise. Previous research has shown that noise reduces phoneme intelligibility and hampers spoken-word recognition – especially for non-native listeners. In the present study, we investigated how noise influences lexical competition in both the non-native and the native language, reflecting the degree to which both languages are co-activated. We recorded the eye movements of native Dutch participants as they listened to English sentences containing a target word while looking at displays containing four objects. On target-present trials, the visual referent depicting the target word was present, along with three unrelated distractors. On target-absent trials, the target object (e.g., wizard) was absent. Instead, the display contained an English competitor, overlapping with the English target in phonological onset (e.g., window), a Dutch competitor, overlapping with the English target in phonological onset (e.g., *wimpel*, pennant), and two unrelated distractors. Half of the sentences was masked by speech-shaped noise; the other half was presented in quiet. Compared to speech in quiet, noise delayed fixations to the target objects on target-present trials. For target-absent trials, we observed that the likelihood for fixation biases towards the English and Dutch onset competitors (over the unrelated distractors) was larger in noise than in quiet. Our data thus show that the presence of background noise increases lexical competition in the task-relevant non-native (English) *and* in the task-irrelevant native (Dutch) language. The latter reflects stronger interference of one’s native language during non-native spoken-word recognition under adverse conditions.

## Introduction

The fundamental process that underlies spoken-word recognition is translating continuous acoustic cues into abstract representational units that allow access to meaning. To achieve this feat, listeners must map the incoming speech signal onto abstract phonological representations. A hallmark of theories on native (e.g., Luce & Pisoni, 1998; Magnuson et al., 2020; Norris & McQueen, 2008; Norris et al., 2000) and non-native (e.g., Shook & Marian, 2013) spoken-word recognition is that activation cascades from sub-lexical to lexical levels where words, consistent with the incoming signal, compete for recognition.

Research in the past two decades has motivated what has become the standard view on non-native word recognition, namely that comprehenders experience interference from their native language. The mechanism underlying this behavior is referred to as “non-selective lexical access” (e.g., Dijkstra & van Heuven, 2002; Dijkstra et al., 2019) and stipulates that when comprehending non-native speech, words from the non-native *and* the native language compete for recognition (e.g., FitzPatrick & Indefrey, 2010; Marian & Spivey, 2003; Spivey & Marian, 1999; Villameriel et al., 2022; Weber & Cutler, 2004). Scientists have extensively studied the factors that influence the extent to which listeners experience cross-language interference, including linguistic properties of the incoming speech (Ju & Luce, 2004), listeners’ proficiency in the non-native language (Blumenfeld & Marian, 2007), and the involvement of general cognitive skills, such as executive control (Gastmann & Poarch, 2022).

One aspect that has received considerably less attention is how the presence of background noise influences the competition dynamics in native and non-native languages (but see Fricke, 2022). That is, it is unclear to what extent the native language interferes when recognizing non-native words in noise. Investigating this question is important since speech recognition outside the lab often takes place in noisy environments (Zinszer et al., 2022). Moreover, investigating this question contributes to further unraveling the long-standing observation that problems in understanding speech in noise appear to be amplified in non-native compared to native listening (for reviews, see Garcia Lecumberri et al., 2010; Scharenborg & van Os, 2019).

Research on *native* spoken-word recognition showed that the presence of background noise enhances lexical competition in the native language (e.g., Ben-David et al., 2011; Brouwer & Bradlow, 2015). In line with theoretical frameworks (Mattys et al., 2009, 2012), these authors reasoned that noise reduces the reliability of the perceived speech sounds and that listeners experienced more competition from words, which would – when perceived in quiet – not be considered target candidates.

In two previous studies, we explored the effects of noise on non-native word recognition. Using the visual-world paradigm, where participants’ eye movements to objects are tracked as they listen to input related to a visual scene (for a review, see Huettig et al., 2011a), we found that non-native target word recognition (looks to the picture of a candle as Dutch listeners heard “candle”) was delayed when targets were masked by background noise, reflecting delayed target word recognition (Hintz et al., 2021). Moreover, in a word transcription task, Scharenborg et al. (2018) found that the number of Dutch listeners’ unique misperceptions, an offline measure reflecting the number of words competing for recognition, on hearing an English target increased as the severity of noise on the target increased (see Karaminis et al., 2022, for a computational model capturing this behavior). The results from these two studies are compatible with a phonetically-based account of word recognition in noise where the presence of noise enhances ambiguity in the non-native speech signal, which in turn delays non-native target word recognition and increases the number of misperceptions. These studies did not, however, directly assess whether the presence of noise increased or decreased interference from the native language.

A growing body of studies on bilingual language production has demonstrated that speakers deploy top-down mechanisms to down-regulate the engagement of the task-irrelevant language in the service of focusing attention to the language relevant for the current task (Declerck et al., 2021; Green, 1998; Jackson et al., 2001; Kang et al., 2020; Wu et al., 2020). This literature demonstrates the flexibility with which language users accommodate task-induced challenges. Since comprehending speech in noise is an effortful task, which – compared to speech in quiet – increases the demands on cognitive resources (Zekveld et al., 2011), one may conjecture that down-regulating the engagement of the task-irrelevant native language when recognizing non-native words in noise is particularly beneficial. Such a suppression mechanism also fits well with findings from research on bilingual reading showing that executive control skills influence the extent of cross-language co-activation (Pivneva et al., 2014) and that readers dynamically adjust the accessibility of task-relevant and task-irrelevant languages based on the context in which reading takes place (Hoversten & Traxler, 2020).

Taken together, it is unclear how the presence of background noise affects interference from the native language during non-native word recognition. Based on the literature, two accounts emerge: The first phonetically-based account predicts that noise increases the ambiguity in the speech signal (Mattys et al., 2009, 2012). In line with models of non-selective lexical access, enhanced ambiguity is likely to increase lexical competition in the non-native *and* the native language (Ben-David et al., 2011; Brouwer & Bradlow, 2015). Thus, this account predicts an increase in cross-language interference. The second account capitalizes on the deployment of top-down mechanisms, which suppress the engagement of the native language during non-native comprehension to allocate resources to the task at hand. This account predicts that the presence of background noise reduces cross-language interference.

## The present study

We tested these accounts using a variant of the visual-world paradigm. We measured Dutch participants’ eye movements as they listened to English sentences containing a target word while looking at sets of four pictures on the computer screen. On target-present trials, one object depicted the target word and the other three were unrelated distractors. On target-absent trials, the target word was not depicted and, instead, the display contained an object whose English name overlapped with the English target in phonological onset. Moreover, the display contained an object whose Dutch (but not English) name overlapped with the English target in phonological onset. The remaining two pictures were unrelated distractors. Tracking participants’ eye movements to target and competitor objects provided us with a time course of how quickly they recognized the non-native targets and whether/when they experienced competition in the non-native and their native language. To test how the presence of noise affects (cross-language) competition, we presented trials either in quiet or masked by speech-shaped background noise.

Compared to speech in quiet, we predicted that noise delays the fixation biases for the target objects, reflecting delayed target word recognition (Hintz et al., 2021). Moreover, in line with the offline transcription data from Scharenborg et al. (2018), we expected enhanced lexical competition in the non-native language in noise compared to speech in quiet, reflected in more looks to the English competitors (compared to the unrelated distractors) in noise. Crucially, comparing fixations to the Dutch competitors across quiet and noise conditions should distinguish between “phonetically-based” and “language suppression” accounts. If noise enhances the ambiguity in the non-native speech signal, which in turn engages a larger set of non-native *and* native competitor words, there should be more looks to the Dutch competitors in noise than in quiet. If participants deploy top-down mechanisms to down-regulate the engagement of their native language to allocate resources to the task-relevant non-native language, there should be fewer looks to the Dutch competitor in noise than in quiet.

## Method

### Participants

The sample size was set a priori. We recruited 36 participants for the present experiment, which was similar to previous relevant studies (e.g., *n* = 20, Weber & Cutler, 2004; *n* = 14, Marian & Spivey, 2003, *n* = 12, Spivey & Marian, 1999). One participant stopped after a couple of trials into the experiment, leaving data from 35 participants (24 female, 11 male; mean age = 24.8 years, *SD* = 3.16, range = 20–36 years) for the analysis. All were native speakers of Dutch who had received formal English instruction at school for at least 8 years. Since Dutch television and cinemas present English series and films in their original language (with Dutch subtitles), it is likely that our participants were exposed to English speech even before they received formal instruction.

Participants’ English language proficiency was assessed using LexTALE (Lemhöfer & Broersma, 2012). In this test, participants carried out an un-speeded lexical decision task. On a scale where 50% accuracy reflects chance level and 100% approximates native-like performance, our participants scored on average 84% (*SD* = 11.01, range = 55–100%), demonstrating advanced English comprehension skills. None of the participants reported a history of developmental or acquired speech problems, or a history of hearing or cognitive problems. All participants had normal or corrected-to-normal vision. The study was approved by the ethics board of the Faculty of Social Sciences at Radboud University.

### Materials

Twenty-two quadruples of words were selected for target-present items. Each target-present item consisted of a target word (e.g., *towel*) and three unrelated distractors (e.g., *lion*, *soap*, *butter*). Quintuples of words were selected for 22 target-absent items (see Appendix). The number of items was comparable to previous studies (e.g., *n* = 20, Weber & Cutler, 2004; *n* = 10, Marian & Spivey, 2003, Spivey & Marian, 1999). Each set consisted of a target word (e.g., *wizard*), an English (e.g., *window*) and a Dutch (e.g., *wimple,* “pennant”) phonological onset competitor, and two unrelated distractors (e.g., *jeans*, *bike*). None of the competitor or distractor words were semantically related to each other or to the target. While the English and Dutch onset competitors overlapped with the target in phonological onset, their Dutch and English translations, respectively, did not overlap phonologically with the target, with each other, or with any of the distractors. The distractors did not phonologically overlap with the targets. Table 1 summarizes the word properties of the target-absent items. A one-way ANOVA for word frequency (*F*(4,105) = 0.70, p = 0.59) and two chi-square tests based on loglinear regression models for number of phonemes (χ^2^(4) = 2.47, p = 0.65) and phoneme overlap with the target (χ^2^(1) = 0.04, p = 0.84), respectively, confirmed that there were no significant differences among the words.

**Table 1:** Word properties of target-absent items

Word	No. of phonemes	Frequency^1^	Onset overlap with target (no. of phonemes)
Target	M: 4.27 (1.16) range: 3–7	M: 4.25 (.56) range: 3.28–5.35	–
English onset competitor	M: 4.18 (1.14) range: 3–7	M: 4.17 (.46) range: 3.57–5.37	M: 2.18 (.39) range: 2–3
Dutch onset competitor	M: 4.86 (1.52) range: 3–8	M: 3.67 (.58) range: 3.00–4.73	M: 2.09 (.43) range: 1–3
Distractor 1	M: 4.23 (.97) range: 3–7	M: 4.18 (.55) range: 3.38–5.02	–
Distractor 2	M: 3.91 (1.02) range: 3–6	M: 4.41 (.53) range: 3.33–5.48	–

^1^As suggested by van Heuven et al. (2014), raw word frequency counts were transformed into Zipf values, calculated as log (frequency of occurrence per one million words) *+ 3*

**Fig. 1:**
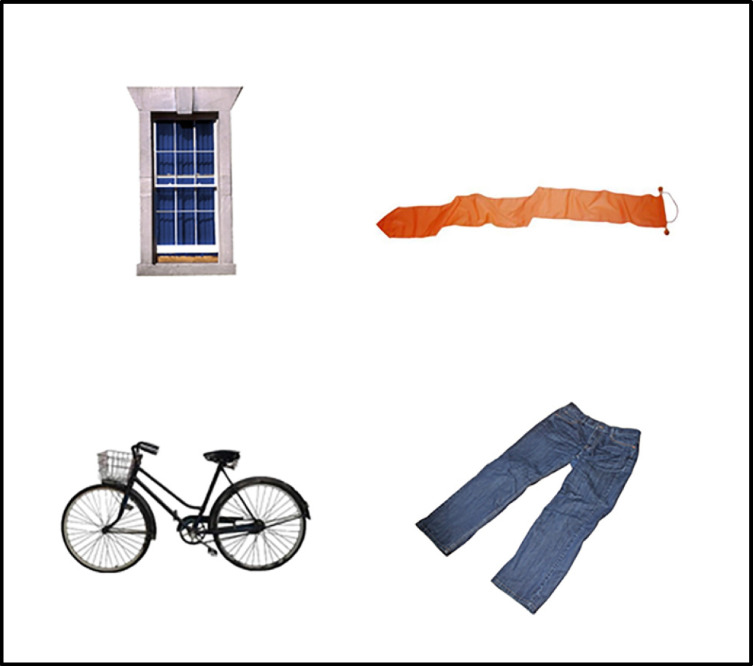
Example of a visual stimulus used on target-absent trials. For the spoken sentence, *It was hard to read, but the note had the word ‘wizard’ written on it*, the display consisted of photographs of a window (English phonological competitor), a pennant (Dutch: *wimpel,* Dutch phonological onset competitor), a bike, and jeans (both unrelated distractors)

All words in the target-absent and target-present items were picturable. Pictures for the target, the two onset competitors, and the distractors were taken from the databases provided by de Groot et al. (2016) and Brodeur et al. (2010), or were found using an online search engine (see Fig. 1, for an example of a visual stimulus used on target-absent trials).

We conducted two rating studies, similar to de Groot et al. (2016), to assess the semantic and visual similarity in target-absent and target-present items between the concepts invoked by the target words and the four objects in each visual display. The rating studies were necessary to ensure that – except for the overlaps in phonological onset – all objects were unrelated to the target words (as overlaps at semantic and visual levels can affect participants’ gaze patterns; Huettig & Altmann, 2005, 2007).

#### Semantic and visual similarity ratings.

Eleven participants provided semantic similarity ratings and 11 others provided visual similarity ratings. None of these participants took part in the main experiment. In both rating studies, participants read the 22 words used for target-absent and the 22 words for target-present trials, presented in the center of a computer screen. Each target word was paired with the four pictures that constituted the target-absent and target-present displays. Target-absent and target-present items occurred in random order. In the semantic similarity rating, participants were asked to judge meaning similarity while ignoring shape similarity. In the visual similarity rating study, participants were asked to judge how similar the typical visual shape of the concept denoted in the printed word was to the physical shape of the referents of the depicted objects, ignoring any similarity in meaning. A rating scale ranging from 1 (no similarity) to 9 (identical) was used in both tasks. As the object referred to by the written word was among the four pictures in target-present items, we also obtained a measure of how well the object name fitted its visual and semantic representation. The semantic similarity ratings confirmed that the target objects matched the semantic representations invoked by the written target words in the target-present items (mean target rating = 8.60, *SD* = 1.41; average of the three distractors = 1.16, *SD* = 1.06). English (1.33, *SD* = 1.18) and Dutch (1.10, *SD* = 0.55) onset competitors, and the two distractors (1.24, *SD* = 0.68) in the target-absent items were rated to be semantically dissimilar to the target. The results of the visual similarity rating confirmed that the target objects depicted the concepts invoked by the written words in the target-present items (mean target object rating = 8.77, *SD* = 1.12; average of the three distractors 1.51, *SD* = 1.32). English (1.67, *SD* = 1.4) and Dutch (1.35, *SD* = 0.85) phonological onset competitors in target-absent items were rated to be visually dissimilar to the target, as were the two distractors (1.5, *SD* = 1.1).

Target-absent and target-present target words were embedded in neutral carrier sentences, where they could not be predicted from the sentential context (see Appendix). The position of the target words in the sentences varied to make an effort towards more ecologically valid stimuli. Sentences were spoken with neutral intonation at a normal pace by a female native speaker of British English. Recordings were made in a sound-damped booth, sampling at 44 kHz (mono, 16-bit sampling resolution). The mean sentence duration was 3,493 ms (*SD* = 834). Onsets and offsets of all words were annotated using Praat (Boersma, 2001). Spoken words on target-absent trials were on average 412 ms long (*SD* = 119); spoken words on target-present items were on average 384 ms (*SD* = 105) long.

A second version of each recorded sentence was created by adding stationary speech-shaped background noise to all sentences using Praat. The added noise spanned the whole duration of the sentence recording. Informed by two previous visual-world studies in our lab (Hintz & Scharenborg, 2016; Hintz et al., 2021), we chose a signal-to-noise ratio (SNR) of + 3 dB SPL. We aimed for a scenario where the presence of background noise would decrease the certainty with which listeners recognize the spoken targets while not resulting in substantial amounts of misperceptions. Our earlier word transcription study with Dutch non-native listeners of English suggested that speech-shaped background noise masking at + 3 dB, with no pictorial input present, would result in about 90% accuracy for non-native target word recognition (Scharenborg et al., 2018).

### Procedure

Eye movements were tracked using an EyeLink 1000 remote desktop tracker, running on Experiment Builder, sampling at 1 kHz. The quiet and noise-added versions of the 22 target-absent and the 22 target-present items were evenly distributed across two lists (i.e., each list featured 44 trials, 22 quiet and 22 noise-added). None of the target words appeared twice on one list. Participants were tested individually. Each participant was randomly assigned to one list and seated in a sound-shielded booth. They placed their head on a chin rest and the eye-tracker was calibrated. Participants received the following instructions (translated from the written Dutch instructions): “In the present experiment you will see images on the screen, while hearing spoken sentences. Half of the sentences will be presented in background noise; the other half will be presented in quiet. Which half you will hear first is determined by the computer. Your only task is to listen carefully to the sentences and to look at the pictures. Furthermore, it is important that you do not look away from the screen or move your head a lot. Before each sentence, we ask you to fixate on a dot that will appear in the middle of the screen.”. In other words, we used a “look-and-listen task” where participants did not receive a specific viewing/response instruction (see Huettig et al., 2011a, for discussion). Such a task allows the listener to inspect the scene without a set goal in mind. Earlier research showed that look-and-listen tasks yield results comparable to tasks where participants are instructed to click on one of the depicted objects (e.g., Huettig & Altmann, 2005; Yee & Sedivy, 2006). Indeed, competitor effects – captured with active and passive tasks – in the visual-world paradigm can be explained by working-memory accounts of language-vision interactions (Huettig et al., 2011b). Specifically, on this account, listeners are assumed to retrieve the names (e.g., English and Dutch) for each of the four depicted objects during the preview phase and to keep these in working memory. As listeners perceive the incoming speech sounds (e.g., the target), they map the phonological representations they identify onto the phonological codes (i.e., picture names) held in working memory. A match (i.e., partial or full overlap between vision-derived and language-derived phonological representations) increases the likelihood of an eye movement towards the respective objects, which – if sufficient evidence has accumulated – results in a shift in gaze.

The spoken sentences were presented through headphones. A trial was structured as follows: First, a central fixation dot appeared in the center of the screen, followed by a preview of the four objects. The positions of the pictures were randomized across four fixed positions of a virtual 2 × 2 grid (Fig. 1). The playback of the spoken sentences was timed such that preview time on each trial amounted to 3 s before the occurrence of the target word in the sentences. This was done to ensure that participants had enough time to preview the displays and retrieve the four object names. The four objects remained in view for the rest of the trial. Each participant was presented with all 44 trials on one list. Trials were blocked by noise type (i.e., quiet, noise). The order of trials within blocks and the order of blocks were randomized automatically before the experiment. The entire testing session, including informed consent, eye-tracking experiment, and LexTALE, was administered in Dutch and took about 30 min.

### Data analysis

The data from participants’ left or right eye (depending on the quality of the calibration) were analyzed in terms of fixations, saccades, and blinks, using the algorithm provided in the EyeLink software. Fixations were coded as directed to the target, English or Dutch onset competitors, to one of the unrelated distractors, or elsewhere. Data falling within a window starting one millisecond after target word onset and ending at 1,000 ms after target onset were selected for statistical analysis. As the data were collected with 1-ms granularity, this resulted in 1,000 data points per trial.

Participants’ fixations were statistically analyzed using quantile generalized additive models (Fasiolo et al., 2021b; henceforth “qGAMs”). qGAMs are an extension of generalized additive models (“GAMs”; Wood, 2017; see Porretta and Kyröläinen, 2019 for an application to visual-world eye-tracking data). While standard regression methods (which include regular GAMs) consider how predictors affect the *mean* of a dependent variable, qGAMs make it possible to additionally study how effects of predictors (e.g., the presence of background noise) affect different *quantiles* in the distribution of a dependent variable. This approach thus provides a much richer view on the experimental results and enables researchers to examine whether the effects of a given predictor are present/stable across the whole distribution of the dependent variable, or whether the effects are confined to a specific portion in the distribution of the dependent variable (for a discussion, see also Baayen & Smolka, 2020; Tomaschek et al., 2018). Such a statistical approach aligns well with recent demands for increasing the quantification of experimental data (Cumming, 2014).

**Fig. 2:**
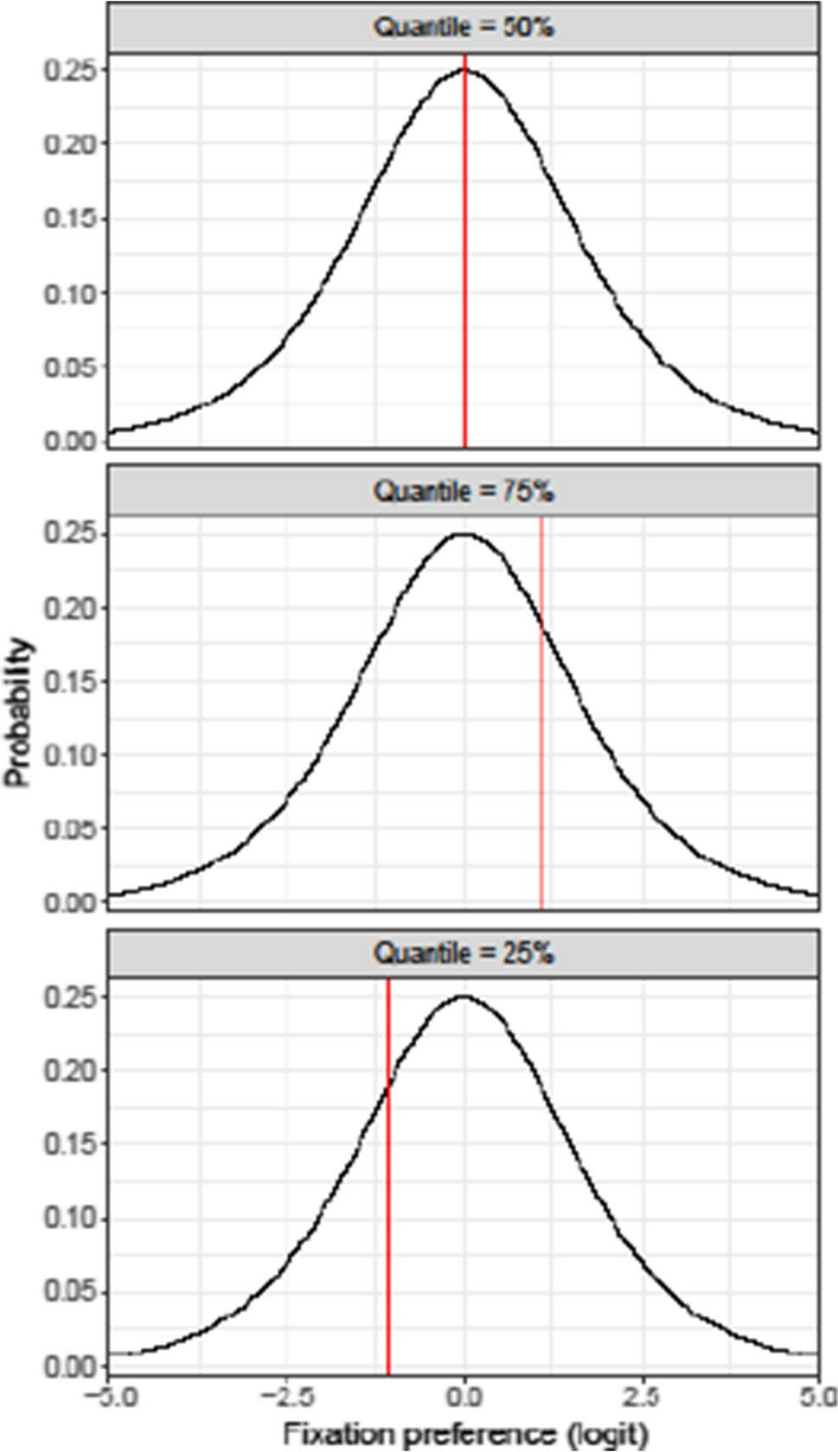
An idealized representation of quantile regression. Each of the three panels shows the distribution of our dependent variable (e.g., fixation preference for Dutch onset competitor). The *x*-axis shows the values that this fixation preference takes in these (simulated) data, whereas the *y*-axis shows that value’s probability of occurrence throughout these entire data. The red lines illustrate the three quantiles that are named in the labels

For an illustration of the logic of qGAMs, consider Fig. 2. The dependent variable (depicted on the *x*-axis) is fixation preference, i.e. the probability of making a fixation to the object of interest (e.g., target, English or Dutch competitor), relative to the averaged distractors. The *y*-axis features hypothetical probabilities of occurrence of data points. The figure consists of three panels. The upper panel illustrates a model fitted to the 50% quantile, which corresponds to the median of the distribution. That is, such a model is optimized for finding effects of predictors (e.g., background noise) that affect the median fixation preference. If fixation preferences are symmetrically distributed, this is equivalent to the *mean* fixation preference, as typically presented and modelled in most analyses of visual-world eye-tracking studies. The middle panel illustrates a model fitted to the 75% quantile of fixation preferences. Such a model is optimized for covering effects that occur at the 75% point of the distribution of the fixation preferences. As the dependent variable in our data is operationalized as fixations to the object of interest *relative* to the distractors, this quantile concerns data points where fixation preferences were larger for the object of interest than for the distractors. Finally, the lower panel in Fig. 2 illustrates a model fitted to the 25% quantile of fixation preferences. This quantile concerns data points that occur at the 25% point of the distribution of the fixation preferences – in our data, reflecting a larger fixation preference for the distractors than for the object of interest.

In sum, while the 50% quantile is comparable to standard approaches of visual-world eye-tracking data analyses that model the mean in the distribution of participants’ fixation behavior, the 75% and 25% quantiles additionally quantify the stability of the effects of listening condition (quiet vs. noise) when considering points in the data distribution when more and fewer looks, respectively, were made to the objects of interest, relative to the distractors.

To analyze the effects of background noise at different quantiles of participants’ fixation behavior, we fitted three types of qGAMs: The first one was fitted to the 50% quantile (i.e., the median) and is comparable to most standard GAM analyses of visual-world data. This analysis was complemented with two qGAMs fitted to, respectively, the 25% and 75% quantiles, to assess whether the effects of background noise change (i.e., across quiet and noise conditions) when considering different portions of the data distribution (i.e., when more and fewer looks, respectively, were made to the target and the competitors in relation to the distractors).

We set up the models as logistic quantile regressions, each fitted to a particular point (viz. a particular quantile) of the distribution of participants’ fixation behaviors. As proposed by Barr (2008), responses were averaged over items. One set of models was fitted for the target-present trials and two sets of models were fitted for target-absent trials – one for the English and one for the Dutch onset competitors. All models compared fixations to the object of interest (target, English competitor, Dutch competitor) to those of the distractors.

In each model, the dependent variable was an indicator coding whether the participant fixated the object of interest (coded as 1) or one of the distractors (coded as 0). An offset term was included, corresponding to the logit-transformed average of the distractors; this has the effect of modifying the chance level assumed in the model from an unconditional 50% to the average of the distractors. As a result, a trajectory estimated by the model to be significantly different from zero will in fact be significantly different from the average of the distractors (i.e., from chance). Smooth terms were added along time for the quiet and noise conditions. Smooth terms are effects (i.e., predictors) in GAMs that use piecewise polynomials to fit, in our case, time, in a potentially non-linear way. This makes it possible to fit temporal trajectories that are more complex than a straight line. A penalty term, estimated automatically via REML, balances the fit between a smooth, straight line and a wiggly curve (a high penalty on the polynomials results in a less complex trajectory; a low penalty results in a more complex trajectory). The technical implementation of our smooth terms was done using the default of thin-plate regression splines, for which we allowed a maximum of 50 basis functions (verified by R function gam.check to be adequate). These splines are the default due to them being provably optimal (Wood, 2017). The target-present models were fitted as described in the previous paragraph; for target-absent models, a sum-coded predictor indicating which of the two onset competitors was being fixated (with appropriate interactions including the smooth terms describing the temporal trajectories of the quiet and noise conditions) was additionally entered into the models.

Models were fitted using the R package qgam (Fasiolo et al., 2021a). Since initial model fits indicated problems due to complete separation,^1^ we followed Donnelly and Verkuilen (2017) in adding a small smoothing constant, which we set to 0.1, to our models. Significance of the terms in the models was established by predicting each model’s linear-predictor matrix onto a grid of time points ranging from 0 to 1,000 ms in both quiet and noise conditions. Using the procedure in Wood (2017: 293–294), 95% Bayesian credible intervals (henceforth: CIs) were computed for the predicted log odds ratios in both quiet and noise conditions, as well as for the difference between these. For the target-absent models, additional differences between the two competitors were computed in the same way. Effects were considered to be significantly above/below chance at time points where their CIs excluded zero.

## Results

### Target-present trials

**Fig. 3:**
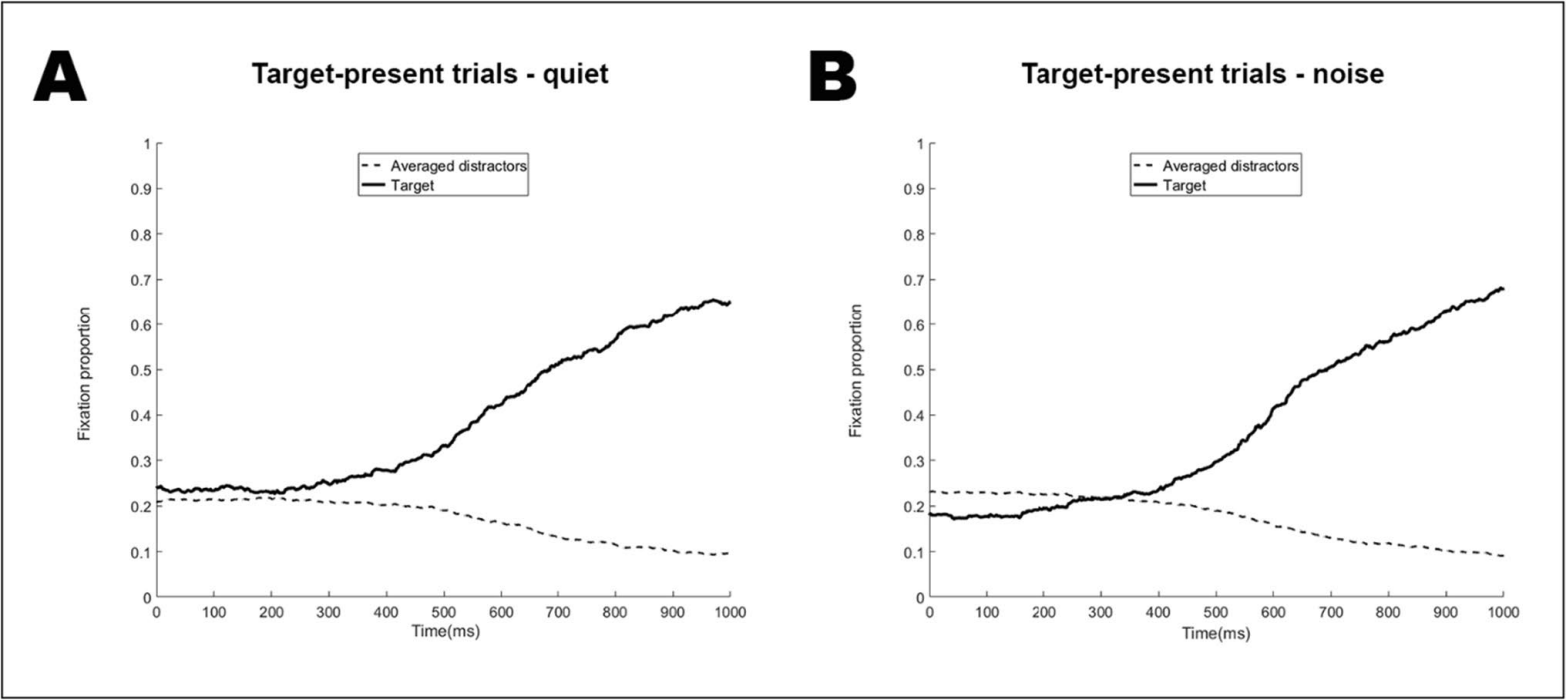
Fixation proportions to target and distractor objects on target-present trials. **Panel A** for the quiet condition, **Panel B** for the noise condition

Figure 3 plots the fixation proportions over time on target-present trials for quiet (Panel A) and noise (Panel B) conditions. In both conditions, participants recognized the spoken targets shortly after spoken onset, as reflected in more looks to targets than to distractors. In the quiet condition, both lines appear to diverge at around 350 ms after onset. Visual inspection suggests that the target fixation bias occurred later when words were recognized in noise, at around 450 ms post-onset.

**Fig. 4:**
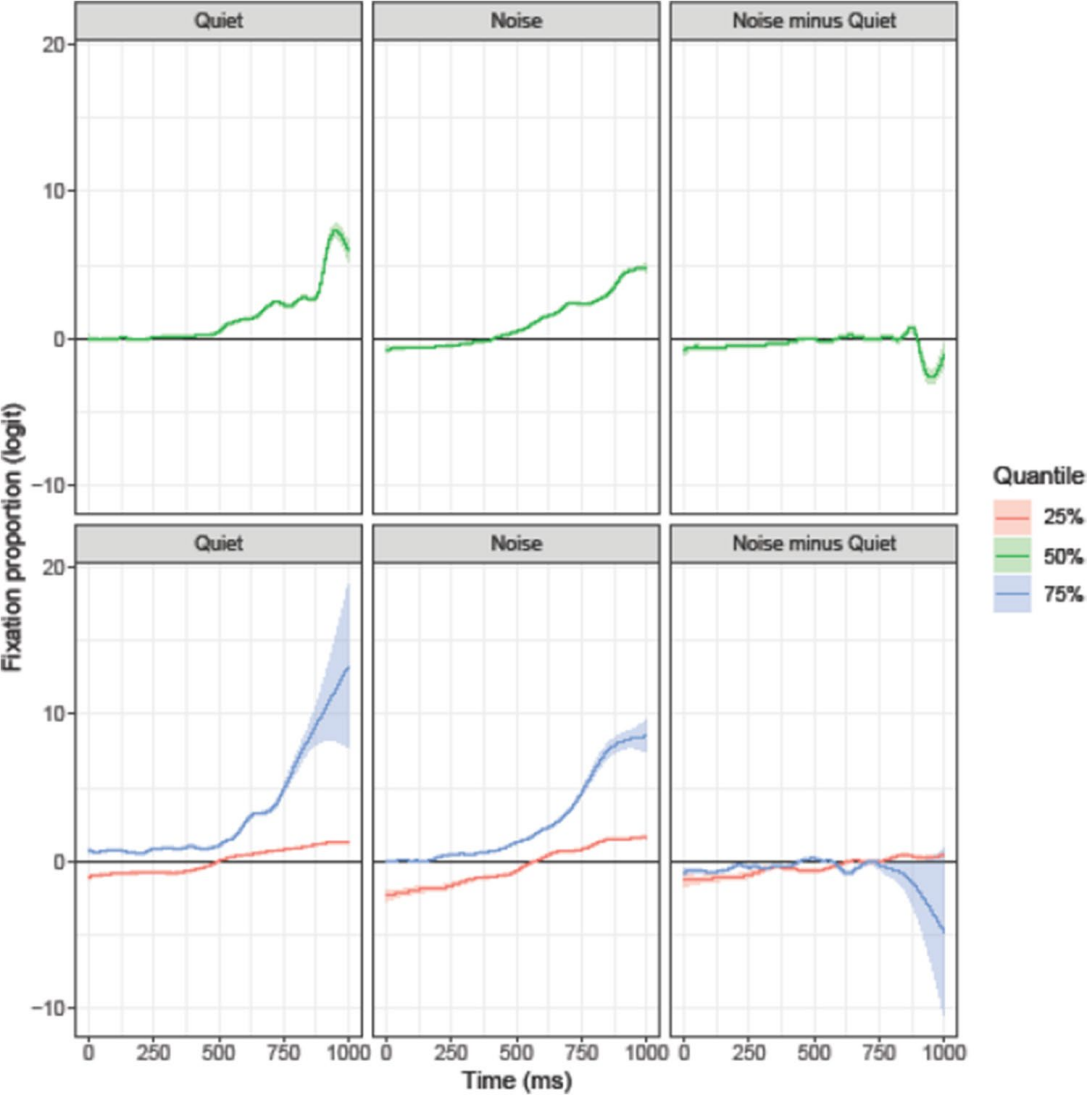
Target-present trials (targets over unrelated distractors). The ribbon indicates the 95% credible interval

Figure 4 shows the modeled temporal trajectory for the target-present trials. The three upper panels feature the results of the 50% quantile (or median, green line) model, which is comparable to the mean that is fitted in non-quantile models. The panels show the fitted fixation preferences in both conditions as well as the difference between them (noise minus quiet). A fixation preference significantly above/below 0 indicates that there were significantly more/fewer fixations to the target than to the distractors. The modelled target fixation trajectories are very similar to the plotted fixation proportions and confirm that in the quiet condition, participants looked more at the target than at the unrelated distractors (log odds ratio larger than 0). This bias reached significance (i.e., CI excluded zero) at 337 ms after target onset and remained significant until the end of the analyzed period. In noise, the bias for the target objects emerged at 427 ms after target onset, and also remained significant until the end of the analyzed period. The upper “Noise minus Quiet” panel in Fig. 4 illustrates the trajectory differences across both conditions. The negative early difference shows that in noise the target bias was reduced compared to the quiet condition.

In the 25% quantile (probability of target fixations was lower than the probability for fixations to the distractors) the target bias reached significance at 507 ms after target onset. In noise, this target bias reached significance at 560 ms after target onset. In the 75% quantile (probability of looks to the target was larger than probability of looks to the distractors), the target bias was significant throughout the entire trajectory in the quiet condition; in noise, it reached significance from 166 ms after target onset. In sum, the analyses of all three quantiles consistently suggest that the presence of background noise delayed and reduced looks to the target objects.

### Target-absent trials: English competitor

**Fig. 5:**
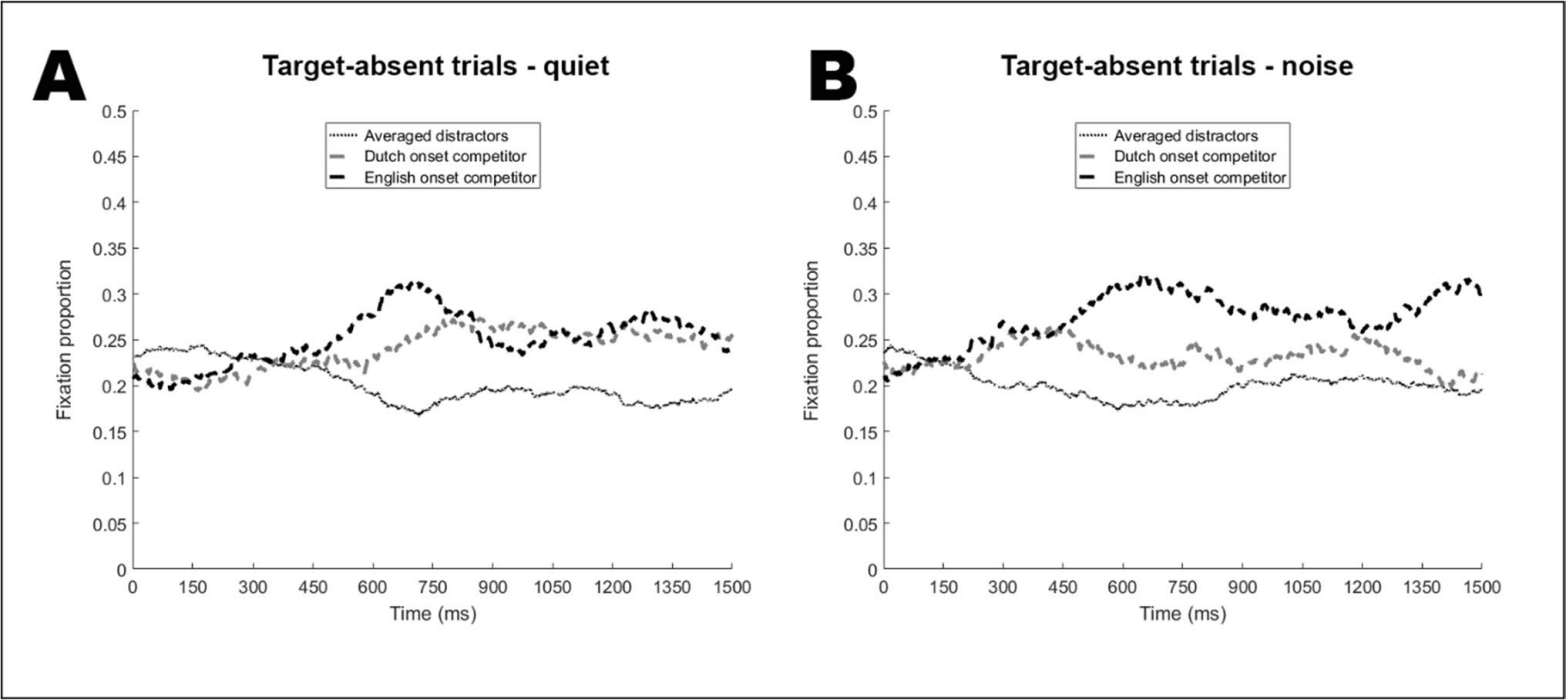
Fixation proportions to English and Dutch onset competitors and distractor objects on target-absent trials. **Panel A** for the quiet condition, **Panel B** for the noise condition

Figure 5 plots the fixation proportions for target-absent trials in the same way as for the target-present trials. The plots suggest that participants displayed fixation biases to both English and Dutch competitors (over the unrelated distractors) in both quiet and noise. While in the quiet condition both biases seem to emerge around 300–350 ms after onset (Panel A), they appear to start substantially *earlier* in the noise condition.

**Fig. 6:**
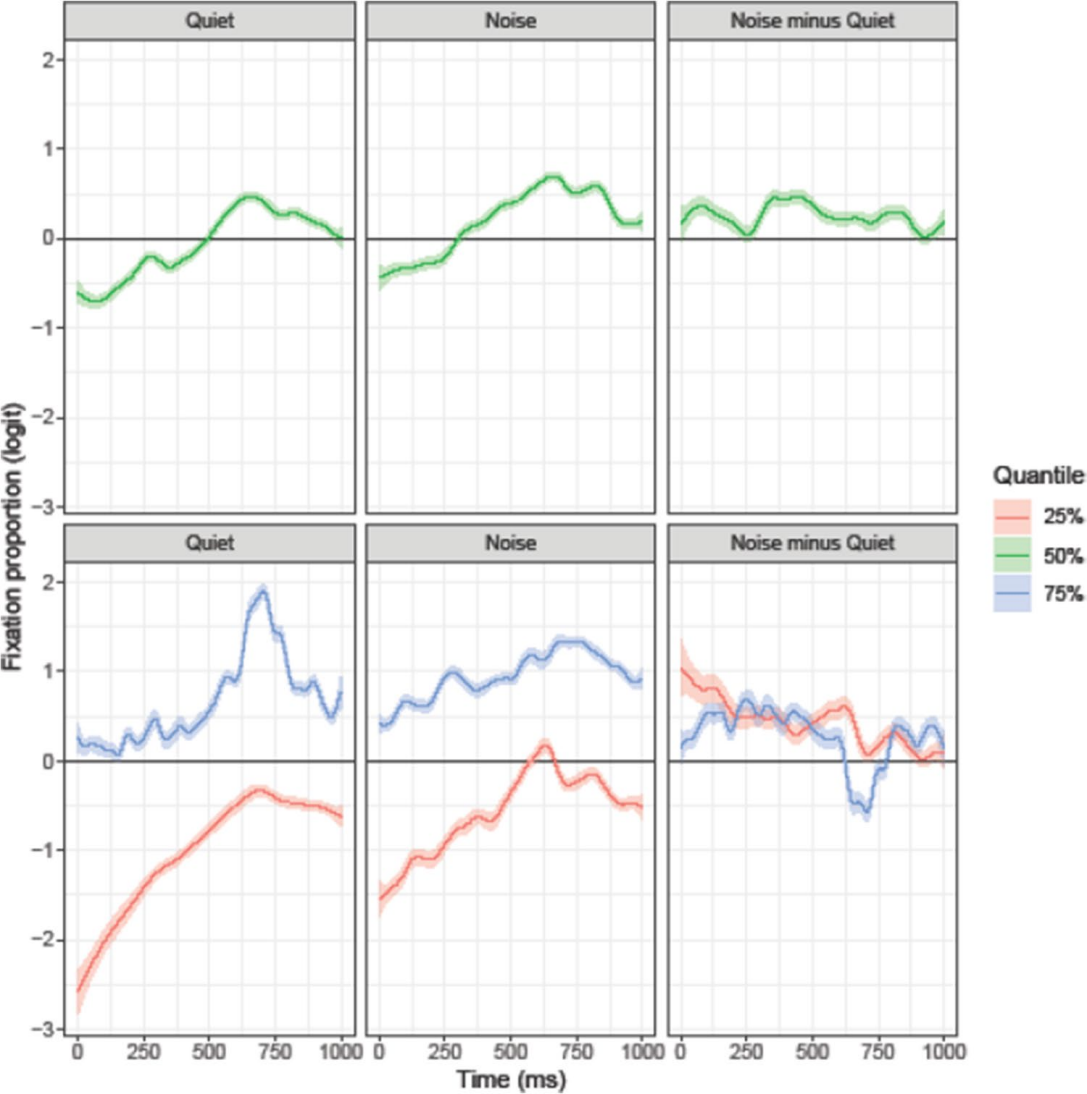
Target-absent items, English competitor. The ribbon indicates the 95% credible interval

Figure 6 shows the modeled temporal trajectories for the English competitor. The results of the median model (green line) confirm the English competitor fixation biases statistically: In the quiet condition, English competitors were looked at significantly more than the distractors from 508 ms after word onset. In noise, the same objects showed an earlier fixation bias starting at 317 ms after word onset. In both conditions, the biases remained significant until the end of the analyzed trajectory. With the probability of fixations to the English competitor reduced (25% quantile), participants did not display a fixation bias for the English competitor object in the quiet condition. In noise, however, there was a brief period (598–657 ms after target onset) where participants favored the English competitor over the distractors. With the probability of fixations to the English competitor increased (75% quantile), participants consistently favored the English competitors over the distractors in both quiet and noise conditions. However, the lower difference plot in Fig. 6 suggests that the fixation bias for the English competitors was consistently larger in noise than in quiet (except for a brief period late in the trial, 629–748 ms). In sum, the analyses of all three quantiles suggest that the presence of background increased the likelihood of looks to the English competitors.

### Target-absent trials: Dutch competitor

**Fig. 7:**
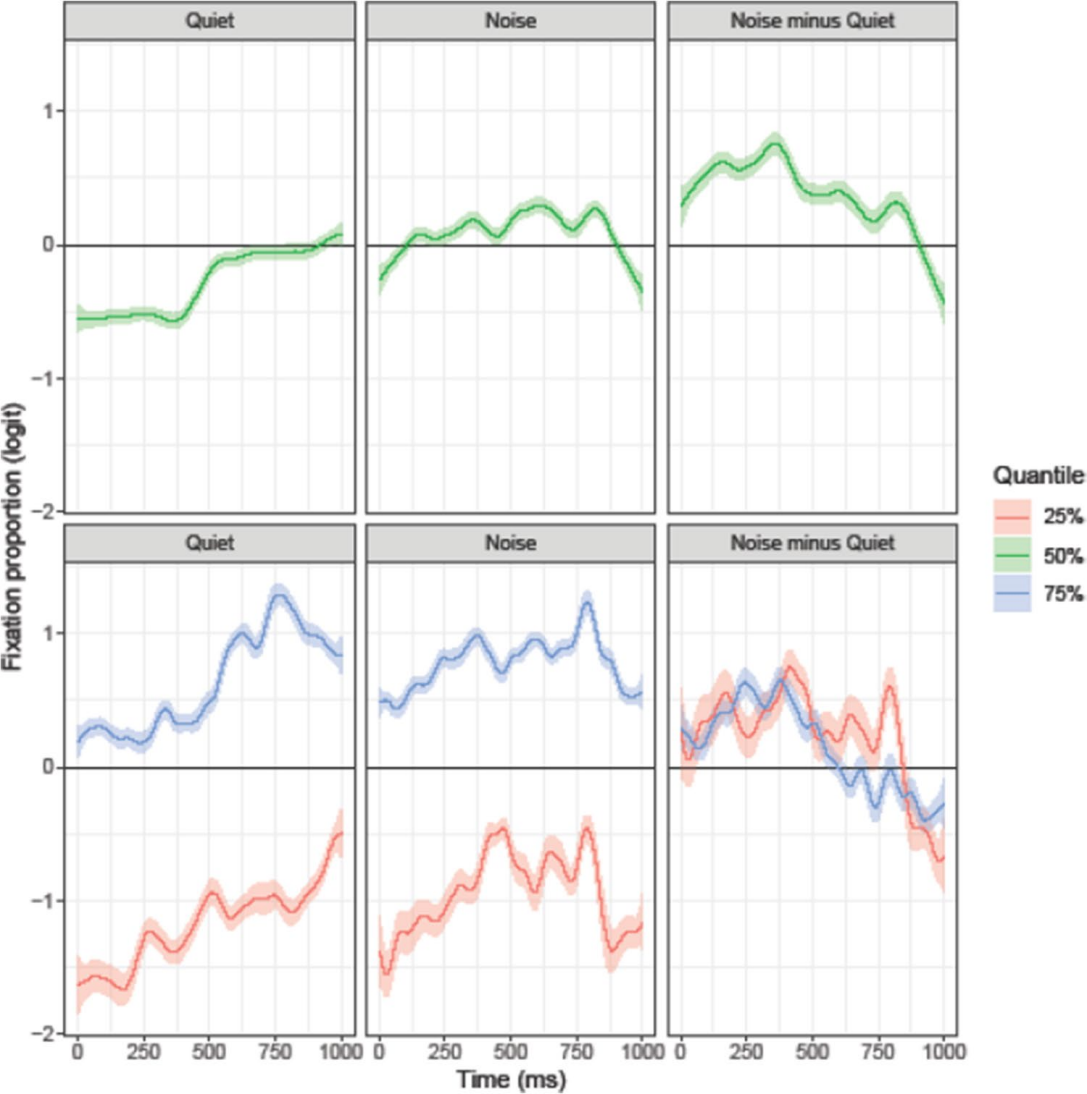
Target-absent items, Dutch competitor. The ribbon indicates the 95% credible interval

Similar to the English competitor, visual inspection of Fig. 5 suggests fixation biases to the Dutch competitor starting at around 450 ms in the quiet and at around 200 ms in the noise condition. However, the results of the statistical analysis (e.g., median model, green line in Fig. 7) show that in quiet, the fixation bias for the Dutch competitors over the distractors reached significance very late, starting 959 ms after target onset. In contrast, the presence of background noise substantially increased the likelihood of looks to the Dutch competitors such that the fixation bias was significant at 129 ms after target onset. With the probability of fixations to the Dutch competitor reduced (25% quantile), there was no significant fixation bias for the Dutch competitor either in quiet or in noise; however, the lower difference plot in Fig. 7 shows that the presence of noise generally increased the likelihood of looks to the Dutch competitor. When considering the 75% quantile, participants consistently looked more at the Dutch competitor than at the distractors in both listening conditions. However, as before the presence of noise resulted in a larger likelihood of looks to the Dutch competitor. In sum, the analyses of all three quantiles suggest that the presence of background increased the likelihood of looks to the Dutch competitors.

### Comparison of English and Dutch competitor fixations

**Fig. 8:**
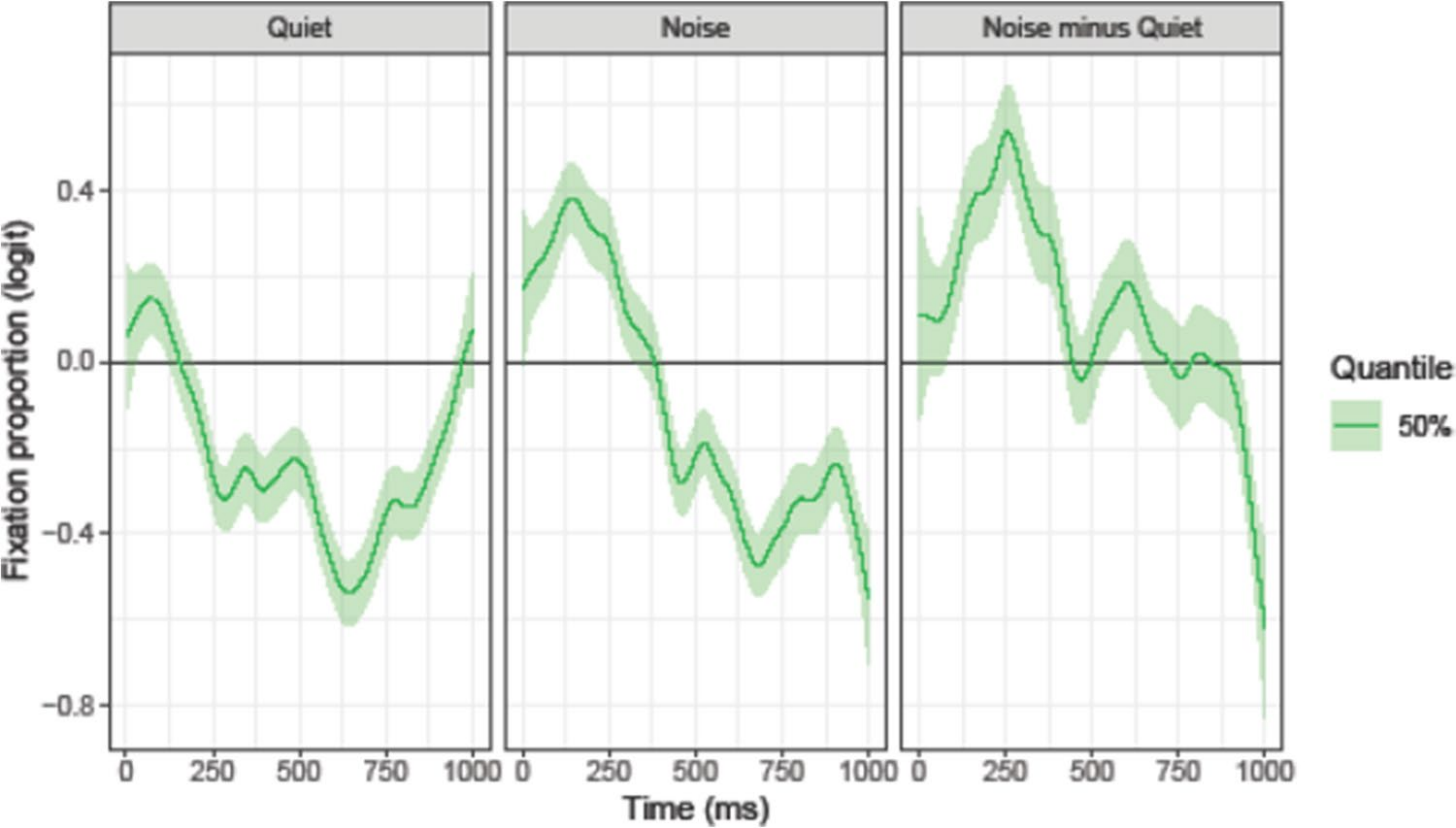
Difference between the Dutch competitor and the English competitor, computed as Dutch competitor minus English competitor. The ribbon indicates the 95% credible interval

Although the results above suggest that the effects of noise on English and Dutch competitors were overall quite similar (i.e., greater likelihood of looks to both competitors in noise than in quiet), we assessed differences in the time course in fixation behavior in a direct comparison. Figure 8 provides the results of this comparison, obtained by computing the estimated trajectories for the Dutch competitor minus those for the English competitor, estimating the corresponding 95% CIs in the usual way. If the biases (i.e., the differences between the respective competitor and the distractors) did not differ, the trajectory should be zero throughout the analyzed period. A negative difference reflects a preference for the Dutch competitor and a positive difference a preference for the English competitor. For the sake of simplicity, this analysis focused on the 50% quantile. The analysis revealed that in both listening conditions there was initially a larger preference for the Dutch competitor. In the quiet condition, this bias reversed at 190 ms after target word onset. In noise, the same general pattern was observed, but the reversal between the competitor preferences occurred later, namely at 401 ms after target onset. Importantly, the difference panel “Noise minus Quiet” in Fig. 8 shows that the difference in fixation behavior across listening conditions was larger for the Dutch competitor: Compared to the English competitor, the likelihood of looking at the Dutch competitor (rather than the distractors) was larger in the presence of background noise than in quiet. In sum, the comparison of fixation biases for English and Dutch competitors suggests that there was initially a larger likelihood for a fixation bias for the Dutch than for the English competitor, which, however, later reversed. The presence of background noise increased the likelihood for a fixation bias for the Dutch competitor more than it did for the English competitor.

## Discussion

Our results suggest that the presence of background noise delays target word recognition, as reflected in later and fewer looks to the target objects in noise compared to quiet – replicating earlier research (Hintz et al., 2021). Whereas noise delayed non-native target word recognition, it resulted in more fixations to the English competitors, reflecting enhanced non-native lexical competition. This finding is in line with the transcription data by Scharenborg et al. (2018), who reported an increase in unique misperceptions as noise intensity increased. Note that this asymmetry (delayed target word recognition and enhanced lexical competition) rules out accounts where participants were simply more engaged with the task when listening in noise compared to quiet. On such accounts, the target bias should be earlier/larger in noise than in quiet, too.

In terms of cross-language interference, we found that noise resulted in earlier and more looks to the Dutch competitors, compared to the speech-in-quiet condition. This finding is similar to that concerning the English competitor and is well in line with the phonetically-based account outlined above: The presence of noise reduces speech intelligibility and leads to larger ambiguity, especially when perceiving non-native speech sounds (cf. Broersma & Cutler, 2011; Cutler et al., 2004; Hazan & Simpson, 2000). Larger ambiguity in turn enhances lexical competition (cf. Ben-David et al., 2011; Brouwer & Bradlow, 2015) as words compete for recognition that might in quiet not be considered target candidates. Crucially, our data suggest that the competitor space widened for both non-native *and* native target word candidates – a pattern generally predicted by models that assume non-selective lexical access (e.g., Shook & Marian, 2013).

Enhanced cross-language interference in the presence of background noise rules out an account where listeners deploy top-down mechanisms to “globally” suppress the engagement of their native language. One might even argue that the opposite is the case. As revealed by our comparison of the time course of English and Dutch competitor fixations, in both listening conditions participants showed an initial preference for looks to the Dutch competitor, which – after some time – flipped to a preference for the English competitor. Interestingly, while the reversal occurred at around 200 ms in the quiet condition, it occurred at around 400 ms in the noise condition. One interpretation of this pattern (complementary to the phonetically-based account) is that non-native listeners have an initial preference for mapping incoming speech sounds onto native mental representations (possibly due to more extensive experience in native-language processing; Krizman et al., 2017). Under optimal listening conditions, high-proficiency non-native language users as in the present study readily suppress their native language in favor of the language relevant for the current task. However, under more effortful conditions, where listeners are burdened with enhancing the relevant (i.e., speech) and suppressing the distracting (i.e., noise) signal, suppressing cross-language interference might be compromised (i.e., delayed). Note that this interpretation relates to that by Hoversten and Traxler (2020), who argued that the context in which language processing takes place shapes the accessibility of native and non-native languages. Clearly, more research is needed to further test this conjecture.

To conclude, we have shown that non-native listeners experience larger interference from their native language when recognizing non-native words in noise compared to quiet. This finding is likely an important contributor to the enhanced difficulties that non-native listeners experience when recognizing words in noise.

## Data Availability

The data and R script for this experiment are available on the Open Science Framework (https://osf.io/6twja/). The experiment was not was preregistered.

## Appendix

Table

**Table 2:** Target-absent items

Carrier sentence containing target word	EN competitor	DU competitor	Distractor 1	Distractor 2
It looked like a *bag*, but much smaller	bat	berg (mountain)	cable	painter
He had trouble spelling the word *'bandage'*, so he asked for help	backpack	bergtop (peak)	wine	sailor
He wandered around and saw the *bowl* he was looking for	bone	boom (tree)	dress	tent
He asked her if she knew another word for *brim*	brick	bril (glasses)	sausage	ski
During the drawing game, she drew a *carrot*, and it was guessed right away	carriage	ketting (necklace)	toilet	mattress
Because he is dyslectic, he read the word *'carving'* wrong	casket	kapstok (hat rack)	peanut	roof
He suddenly saw the *cattle* appearing in the dark	cap	kers (cherry)	monkey	snow
The girl thought about the meaning of the word *'danger'* after her father's story	daisy	deken (blanket)	fish	pen
She recently saw a picture of a *dentist* on the internet	desert	deksel (lid)	shirt	pencil
He recently bought a painting of a *dog* from a famous artist	dot	dop (pod)	stamp	train
He asked her about the spelling of the word *'lobster'*, because he did not know it	locker	lorrie (trolley)	flute	orange
He had never seen the *lock* before because it was well hidden	log	lont (fuse)	hammer	apple
We left the house, but forgot the *map* with all directions	match	mes (knife)	turtle	bed
The dictation contained the word *'mayor'*, but nobody had it wrong	maple	meetlint (measuring tape)	cello	smoke
Would you know the word for *'parlour'* in Dutch?	party	patat (chips)	hippo	nail
Finally, we found the *pillow* that my little brother wanted so badly	pigeon	pinpas (debit card)	jacket	honey
The girls seemed surprised about the *rain* that did not stop for days	rail	reep (bar)	nurse	mouse
They were talking about the *soccer* game that they saw	saucer	sokkel (pedestal)	trumpet	laptop
She laughed about the story that the *teacher* told us last week	stomach	stormram (battering ram)	llama	table
He was quite excited about the *wallet* he was just given	wardrobe	wokkel (twist)	mouth	ball
The girl did not know how to spell the word *'wink'* during the exam	wing	wip (skip/seesaw)	mango	thermos
It was hard to read, but the note had the word *'wizard'* written on it	window	wimpel (banner)	jeans	bike

2

## Footnotes

1 “Complete separation” refers to a condition that prevents the convergence of the maximum likelihood estimation for the coefficient(s). This can be the case when a linear combination of the predictors yields a perfect prediction of the response variable.
